# The Genetic Diversity of African Common Bean Germplasm: A Systematic Review of Reported Molecular Studies

**DOI:** 10.3390/genes17010075

**Published:** 2026-01-09

**Authors:** Tatenda Ephraim Chikasha, Rogerio Marcos Chiulele, Wilson Nkhata, Bernado Lazaro Muatinte

**Affiliations:** 1 Plant Production Department, Eduardo Mondlane University, Av. Julius Nyerere, nr. 3453, Maputo 1100, Mozambique; tatendachikasha@uem.ac.mz (T.E.C.);; 2 International Centre for Tropical Agriculture, Chitedze Agricultural Research Station, Lilongwe P.O. Box 158, Malawi; 3 Biological Sciences Department, Eduardo Mondlane University, Av. Julius Nyerere, nr. 3453, Maputo 1100, Mozambique

**Keywords:** *Phaseolus vulgaris* L., genetic diversity, population structure, molecular markers, Southern Africa, breeding programs, conservation

## Abstract

**Background**: Common bean (*Phaseolus vulgaris* L.) is an important grain legume crop of nutritional and economic value across Africa. Genetic improvements of the crop to enhance productivity and resilience depend on understanding the diversity within the African germplasm. **Methods**: Following PRISMA guidelines, the genetic diversity and population structure of common bean in Africa were reviewed systematically based on existing research. A protocol for conducting the systematic review was developed registered in OSF. Twenty-nine studies met the inclusion criteria after a comprehensive search in ScienceDirect, PubMed, Google Scholar, PubMed, AGRICOLA, Taylor & Francis, and SpringerLink. Data on molecular markers and diversity metrics, thus PIC, He, and AMOVA, were extracted and synthesized qualitatively. **Results**: Despite substantial heterogeneity in panel sizes, reporting completeness, and marker systems (SSR, SNP, POX, ISSR), consistent patterns emerged. Studies revealed moderate to high levels of genetic diversity. Population-structure analyses recovered the canonical Andean and Mesoamerican gene pools with extensive admixture and high gene flow. AMOVA results indicated that a substantial proportion of total genetic variation was attributed to within-population components. **Conclusions**: The results are consistent with previous studies, but the sample size and types of markers make direct comparisons impossible. More future studies should use standardized genotyping approaches to increase data consistency. These insights are useful for yield improvement under both non-stress and stress conditions and for developing Africa’s diverse environments.

## 1. Introduction

Common bean (*Phaseolus vulgaris* L.) stands as a fundamental legume crop throughout the world since it offers crucial dietary content. It serves protein alongside vitamins, minerals, and fiber to millions of low-income families throughout Africa, Latin America, and developing regions [1,2,3,4]. *P. vulgaris* exhibits broad adaptability, with documented cultivation spanning diverse agro-ecological zones. This covers from tropical lowlands (<1000 masl) to high-altitude Andean regions (>2000 masl), and a range of temperature regimes from 18 °C to 30 °C during growing seasons [5,6,7]. Common beans’ short life cycle and their rich nutritional profile make them both a dietary basis and a reliable source of revenue for small-scale farmers [5,7]. However, common bean production in Sub-Saharan Africa remains below 200 kg per hectare compared to the worldwide cultivation standard of 2000 kg per hectare [6,8]. Major constraints stifling common bean production include biotic factors (pest infestations, pathogen pressure, parasitic weeds) and abiotic factors (soil factors, climate variability, extreme weather events, and photoperiod sensitivity). These stressors drive yield instability [5,9,10], thereby undermining the importance of the crop in the region. Therefore, urgent attention must be directed towards creating stress-tolerant and high-yielding cultivars of the crop. The strategic exploration of the extant genetic diversity through modern genomic tools remains fundamental to these breeding objectives [5,9,11].

Common bean is genetically diverse and was derived from two distinct groups, the Mesoamerican and the Andean pools, domesticated in Central and South America, respectively [12,13,14]. Seed size, phaseolin protein patterns, and molecular marker expression of the two pools are different [15,16,17]. Mesoamerican bean varieties are small-seeded, whilst the Andean bean varieties are large-seeded [12,15,17]. Common bean first arrived in Africa during the 16th and 17th centuries, and continual farmer selections gave rise to landrace varieties with geographical origins in Africa [14]. The African continent is globally recognized as a secondary centre for genetic diversity of the common bean, where landraces have unique characteristics in natural gene exchange and environmental adaptation [12,15]. This is documented through nucleotide diversity (π = 0.023–0.028) exceeding primary Mesoamerican gene pools (π = 0.019) and altitudinal adaptation up to 2200 masl [14,18]. These accessions demonstrate unique genomic signatures of environmental selection, particularly drought-responsive *PvP5CS1* alleles and heat-shock protein haplotypes absent in Andean germplasm [14,18].

Such genetic diversity needs to be understood if breeding programs designed to improve yield, stress tolerance, and nutritional quality are to succeed. The use of molecular markers has evolved largely from early technologies (RFLPs, RAPD, AFLP, SSR) to modern-day technologies, including the single-nucleotide polymorphisms (SNPs) [19,20,21]. SNPs, widely adopted since the mid-2010s, are essential in high-resolution analysis of the genetic diversity, population structure, and gene flow assessment [19,20,21]. This molecular marker technology is advantageous because of its reproducibility, and it has a full genomic scope [21,22,23]. Moreover, SNP markers are compatible with high-throughput marker-assisted strategies, which make them suitable for genetic research and selection programs [22,23]. Although these technologies exist, the genetic diversity of common bean in Africa is largely underexplored, lacking the characterization of local landraces and their breeding potential [20,21,24]. Currently, there is a knowledge gap that restricts the development of superior cultivars that would suit specific environmental and economic conditions.

This systematic review evaluated published molecular studies on common bean genetic diversity and population structure conducted in Africa. The objective was to summarise reported patterns of within- and among-population genetic variation, and to examine how differences in marker systems, sampling strategies, and geographic coverage influenced the diversity metrics reported across studies. The review focused on synthesising evidence from heterogeneous datasets without quantitative pooling. Particular attention was given to reported estimates of heterozygosity, population differentiation, and population structure.

## 2. Materials and Methods

### 2.1. Scope of the Review

This systematic review adhered to the PICOTS framework to investigate the genetic diversity of *P. vulgaris* in Africa. The population included all African germplasm, from landraces to improved lines [22,25]. The intervention was defined as any molecular or phenotypic assessment of diversity, aligning with standard genetic characterization methods [20,21]. Outcomes of interest were standard population genetics metrics, such as heterozygosity and FST statistics, to facilitate structured comparisons across studies [26,27]. The scope was deliberately inclusive of all publication years and African sub-regions to ensure a comprehensive analysis [21,25,28]. This approach captured trans-boundary gene flow and farmer-mediated seed systems [21,22] while preventing regional bias.

### 2.2. Review Protocol and Reporting Standards

This systematic review was conducted in accordance with the PRISMA guidelines and followed a protocol registered prospectively in Open Science Framework, Registration DOI: 10.17605/OSF.IO/R2ZA6. This was done to ensure transparency in study selection, data extraction, and synthesis.

### 2.3. Search Strategy

For literature search, this study used Google Scholar, ScienceDirect, Taylor & Francis Online, SpringerLink, and PubMed, all have articles on genetics, plant breeding, and agriculture [29,30]. Supplementary searches included citation tracking of relevant literature [31]. The search also included grey literature sources, such as reports, surveys, and unpublished data [29]. The search was broad in terms of time span to cover both older and newer studies. In building up this comprehensive base for studying the genetic diversity of common beans in Southern Africa, the study followed the best practices of both agricultural and genetic research by using all these sources combined [32,33].

Boolean logic, phrase searching, and truncation were used to find the most relevant studies. These approaches guaranteed inclusion of all important studies [29]. The selection process for the main search keywords involved both synonyms relating to the area of genetics research as well as alternate phrasing options. Some of the keywords were ‘common bean genetic diversity’, ‘*P. vulgaris* germplasm’, ‘Mesoamerican and Andean gene pools’, and ‘molecular markers in common bean’. Then, to collect variations for all possible genetic diversity, genetic markers, and genetics, truncation and wildcard were also used.

Boolean operators narrowed searches by using AND to combine key terms for specificity, for example, “genetic diversity AND *P. vulgaris* AND Africa”, and OR for related concepts, such as “common bean OR *P. vulgaris*”. Studies outside the region were excluded by the use of the NOT operator [33]. The phrase search made sure that terms like ‘Southern Africa common bean’ and ‘genetic diversity assessment’ resulted in exact matches, and created more specific results using the adjacency operators [29]. Insofar as possible, the vocabulary was controlled. MeSH terms like “Legume Genetics” and “Phaseolus Genetic Variation” were included in the search in PubMed [34]. ScienceDirect’s indexing system further improved search precision. To find more studies that cited key publications in the field, citation tracking in Google Scholar was used [31]. Studies were limited to English language publications without date restrictions so that the most recent as well as historical studies could be included. Recent studies using advanced molecular techniques, such as SNP analysis, were given priority. The search strategy was iteratively improved and tested to achieve a maximally relevant and comprehensive foundation for a systematic review.

### 2.4. Eligibility Criteria

To ensure a comprehensive and focused synthesis of the genetic diversity of the Southern Africa common bean panel, the following criteria were applied.

#### 2.4.1. Inclusion Criteria

The studies were included in order to develop a clear and focused synthesis of the genetic diversity within the African common bean panel; thus, research on *P. vulgaris* germplasm. These studies included landraces, cultivars, or breeding lines originating from African countries. Only research studying the diversity of genes, population structure, gene pool (classified as Mesoamerican versus Andean), or adaptive traits such as drought tolerance and disease resistance was considered. To describe the genetic material, studies included had to use molecular markers such as SNPs, SSRs, or AFLPs, among others. The study included original scientific articles, published by indexed with peer review journals, which were experimental, observational, or comparative, preprints, or reputable technical reports that contain original data. Additionally, these studies needed to provide quantitative genetic diversity measures like allele frequency, heterozygosity, and polymorphism rates, or qualitative descriptors including gene pool affiliation and population clusters. There were no limits on publication dates so that both historical and current data could be captured, but only studies published in English were considered, as translation of the studies was not possible.

#### 2.4.2. Exclusion Criteria

This systematic review excluded studies focusing on *P. vulgaris* and relying on the common bean germplasm from regions outside Africa. Research in which only agronomic or phenotypic traits were analyzed, and no genetic marker analysis was conducted. Likewise, reviews, meta-analyses, opinion pieces, or theoretical models without original data were omitted. The studies that had simply focused on the morphological characteristics without including molecular data or used non-reproducible marker systems were also rejected. Any study with incomplete or insufficient genetic data, such as missing allele counts or unclear sampling methods, was also not included. Non-peer-reviewed sources (save theses) were excluded, unless they provided critical unpublished data.

### 2.5. Grouping Studies for Synthesis

A synthesis was done according to several key factors. The studies were by country to allow for country-by-country analyses as well as to proffer a hint of regional comparison. Then, the studies were categorized by type of molecular marker used (i.e., SNPs, SSRs, or AFLPs) to allow comparison of methodological approaches. Gene pool affiliation was also studied, with studies being differentiated as those focused on the Mesoamerican gene pool and those focused on the Andean gene pool. We then finally grouped studies based on the specific adaptive traits that they investigated, such as drought tolerance and disease resistance. This structured approach ensures the provision of a clear, comprehensive synthesis of the available evidence that can support future strategies of bean breeding and conservation.

### 2.6. Selection Process

The selection process of the study was well structured to include only the relevant and high-quality research to meet the inclusion criteria. Multiple stages of screening of the studies retrieved from Google Scholar, ScienceDirect, Taylor & Francis Online, SpringerLink, and PubMed were completed to remove those studies that did not fit the research focus. Each record and the full text article were examined by two independent reviewers in order to maintain objectivity and reduce bias. There was a discussion in cases of disagreement, and a third reviewer was solicited to reach a final decision. The screening started with titles and abstract reviews. Studies beyond the scope of the studies (i.e., studies focusing on common bean germplasm from outside Africa, or lacking genetic marker analysis) were excluded. Full text review was carried out on those that met the basic criteria and further assessed based on their focus on genetic diversity, population structure, and adaptation traits of common bean in Africa.

### 2.7. Data Collection Process

A systematic process was developed to collect data, which produced accurate and reliable information from the studied research works. Data extraction occurred independently through separate reviewers to promote objective results and minimize mistakes. Discrepancies were resolved through discussion, and where necessary, a third reviewer provided a final decision. The standardized data extraction form guided systematic data collection of specific details such as experimental design, together with sample characteristics, genetic marker types, and diversity measures. The reviewers carefully documented both allele frequencies, heterozygosity levels, polymorphism rates, and gene pool classifications. The organized method of data collection eliminated any crucial points that were missed. The final dataset was cross-checked to ensure that all included studies met the research criteria and provided relevant findings.

### 2.8. Data Items

The study reviewed the data that was used to evaluate the genetic diversity of African common bean germplasm with an aim to understand their breeding potential and ways of conserving them. They were studied about their geographic location, genetic diversity parameters, population structure, and farming-related traits [19,21]. Diversity indices such as PIC, MAF, Ho, He, and FST were also extracted [27]. The study then collected useful information on genetic variations using molecular markers such as Single Nucleotide Polymorphism (SNPs), Simple Sequence Repeats (SSRs), among others [20]. In addition, AMOVA was applied to infer genetic diversity within and between groups, as well as to detect certain genetic subpopulations [35].

#### 2.8.1. Missing Data

Given the variety of methodologies used across the included studies, significant heterogeneity was unavoidable. Marker systems, sample sizes, and reporting formats varied greatly from one study to another. Additionally, the temporal range of the studies contributed to differences in marker detail and informativeness, leading to the absence of some key diversity indices in older datasets. During data extraction, any fields that were not reported were labeled NR, as shown in Appendix A. To handle this heterogeneity, all studies were processed using a predefined extraction template that focused on consistently reported diversity indices (PIC, He, Ho, and F_ST). Studies were included if at least one core metric was available. This method preserved comparability while allowing for maximum inclusion and geographic coverage. Missing values were not imputed; instead, they were transparently documented for each study.

#### 2.8.2. Geographic Limitations

The screening process revealed an uneven distribution of molecular diversity studies across Africa, with some regions yielding no eligible studies. This imbalance did not warrant the application of any regional weighting; instead, all studies that met the inclusion criteria were analysed using the same extraction framework. The uneven geographic coverage was documented as an inherent characteristic of the evidence base. Accordingly, the systematic review avoided analytical procedures that assume uniform regional sampling.

### 2.9. Risk of Bias in Included Studies

All 27 studies in the master dataset were appraised for methodological quality using the ROBINS-I tool Version 2 [36], which is a standardized tool to assess bias across non-randomized studies [37]. According to [36], the five main domains of bias (selection bias, performance bias, attrition bias, detection bias, and reporting bias) can introduce a systematic bias in the study outcome. Two reviewers independently assessed each study as being at high risk of bias, low risk of bias, or some concerns risk of bias. These reviewers worked independently to minimize the risk of bias being introduced into summary statistics. Predefined criteria from ROBINS-I were followed, such as whether studies had appropriate random methods, allocation concealment, complete outcome data, unbiased outcome assessments, and full reporting of the results [37]. This process helped to avoid mistakes and ensured a transparent review process. In fact, the reviewers during the evaluation checked if the intervention delivery was consistent without an additional unintended treatment (performance bias). There was also caution to avoid systematic differences in the participant selection (selection bias), and appropriate handling of missing data (attrition bias) [36]. Further, the reviewers examined whether outcome assessors were blinded to avoid biased measurements (detection bias) and whether all relevant findings were reported with no selective omission (reporting bias) [36]. When disagreements occurred, the reviewers would first hash out their assessments by comparing study methods. If there was no consensus, a third senior reviewer reviewed it and made a decision. The result of this stringent, multi-sequential process was that the system encouraged a fair, unbiased, systematic review approach.

### 2.10. Effect Measures

Because of the variation in experimental protocols and outcome measures across studies, no single standardized effect size could be derived. Therefore, the results were synthesized qualitatively rather than combined quantitatively.

### 2.11. Synthesis Methods

Data extracted from the included studies were summarized using a narrative, comparative approach. This was due to substantial differences in germplasm panels, phenotyping methods, trait scoring systems, and molecular marker platforms. Because of this methodological and reporting variability, calculating a single pooled effect size was not appropriate. Conducting a meta-analysis was not possible because outcome measures and experimental conditions were not consistently reported across studies. Instead, results are presented as a structured narrative synthesis, following PRISMA guidelines for heterogeneous datasets, which helps maintain a clear link from each source to its contribution within the overall synthesis. Findings were grouped and compared based on (i) geographic region, (ii) study design, and (iii) type of molecular diversity assessment.

## 3. Results

### 3.1. Study Selection

**Figure 1:**
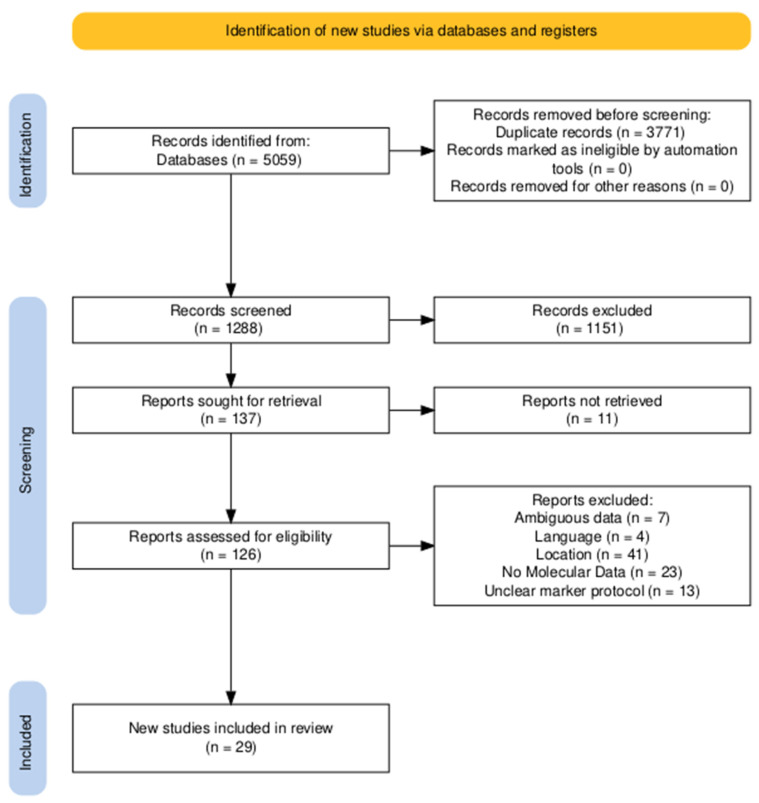
PRISMA flow diagram summarizing study selection process.

The search identified 5059 records across the five sources: Google Scholar (2400), ScienceDirect (2361), Taylor & Francis Online (100), SpringerLink (140), and PubMed (58). Following automated de-duplication in Mendley Desktop (version 1.17.13), 3771 duplicates were removed, leaving 1288 unique records for title- and abstract-level screening. Screening excluded 1151 records that clearly failed to satisfy the preset PICOT criteria; 137 articles were retrieved for full-text assessment. Eleven full texts could not be obtained despite repeated requests to libraries and corresponding authors through the ResearchGate platform, leaving 126 studies for eligibility review. 97 of the studies were excluded at full text for protocol-defined reasons. These included studies conducted outside Africa (*n* = 41), analyses restricted to agronomic or morphological traits with no molecular data (*n* = 23), insufficient or non-reproducible genetic reporting such as missing allele counts/unclear marker protocols (*n* = 13), species or crop mismatch (*n* = 9), duplicate cohorts or overlapping data without added analyses (*n* = 7), and non-English publications without an accessible translation (*n* = 4). The remaining 29 studies met all eligibility criteria and were included in the synthesis. The PRISMA flow diagram (Figure 1) summarizes the identification, screening, eligibility, and inclusion phases with these same counts for full transparency.

### 3.2. Excluded Studies

Firstly, several studies were initially found to fit the inclusion criteria for this review, but were excluded for specific reasons. In [38], the genetic diversity of *P. vulgaris* ecotypes in Pakistan was studied using SSR markers. Although the study was an important data source on genetic variation, it was excluded from this synthesis because it did not focus on the geographic regions of interest in this synthesis, as the studied populations were from Pakistan. Along the same lines, ref. [39] performed a broad analysis of Brazilian common bean landraces by using a Geographic, Agronomic and Molecular Approach (GAMA) to measure genetic diversity and population structure. Although it has an advanced methodology and a large dataset, the study was dismissed, as it refers to a collection of Brazilian germplasm and not the landraces of interest in Africa.

Additionally, the research conducted by [19] examined genetic diversity, linkage disequilibrium, and population structure within a global common bean reference collection. The study was excluded because the authors employed SNP markers for highly detailed genomic assessment, yet focused on worldwide reference collections instead of African landraces. Research conducted by [40] characterized underutilized common bean genetic resources in organic farming fields in two contrasting environments (i.e., suitable and marginal areas). This particular review was related to the assessment of the molecular, agronomic, and nutritional aspects of the plants. It was, however, ruled out because the study was strictly on landraces from Italy, which was out of context for this review. Ref. [41] investigated molecular genetic diversity of Italian common bean landraces with genotyping-by-sequencing (GBS) technology. The study found evidence of hybridization and F3 individuals within the Andean and Mesoamerican gene pools. However, it was excluded because of its geographic focus on Italian landraces, which is not of interest for this review.

### 3.3. Study Characteristics

The studies varied in scope. Some studies analyzed fewer than 50 bean accessions, while others looked at more than 700, covering everything from traditional landraces to modern breeding lines and released varieties. There was also a notable variation in the countries covered and the types of markers applied, as summarized in Appendix A.

### 3.4. Geographic Coverage

**Figure 2:**
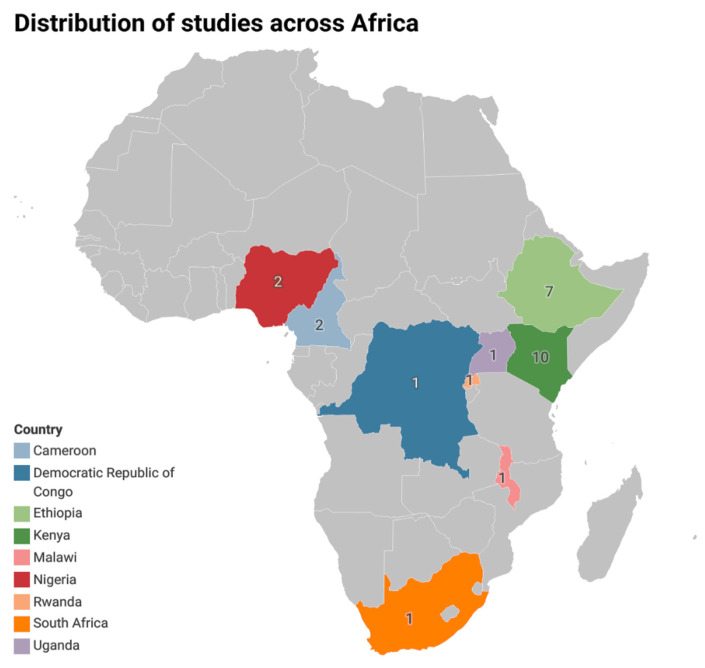
Shows the number of studies conducted per country.

The 29 studies included in this review represented nine African countries, as shown on the map (Figure 2). Research efforts were highly concentrated in Eastern Africa, which contributed 83% of the included studies. This dominance reflected the location of major bean breeding, particularly in Kenya, Ethiopia, and Uganda, supported through the PABRA network and regional CIAT-affiliated facilities. Southern Africa, defined here according to the SADC regional bean network (including DR Congo), contributed three studies, while West Africa (Nigeria) and Central Africa (Cameroon) each contributed two studies. No eligible studies were identified from North Africa. This pattern indicated strong research capacity in Eastern Africa but also highlighted notable geographic gaps, particularly in Southern and West African production zones where productivity remains below par. This pattern is consistent with continental production statistics. Common bean cultivation is concentrated in Eastern and Southern Africa, with more limited production in arid and semi-arid regions.

### 3.5. Marker Technologies

**Figure 3:**
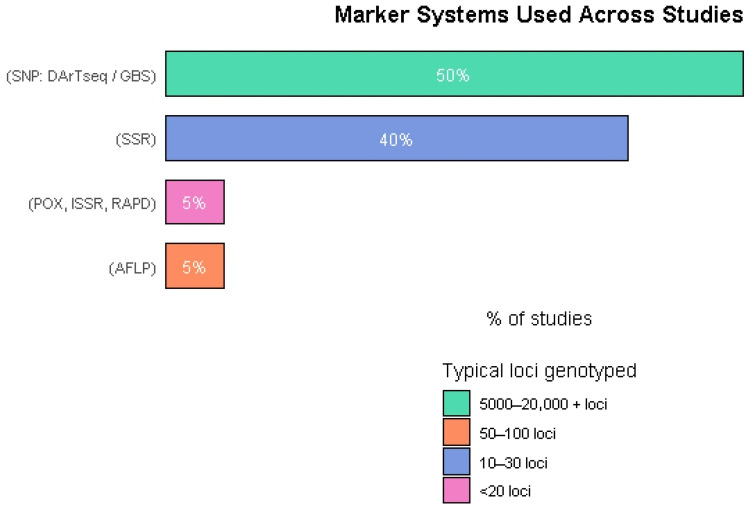
Frequency of molecular marker systems used across the included studies.

Marker technologies used in the studies shifted significantly over time (Figure 3). Early work relied predominantly on SSR markers, valued for their informativeness but limited in genome coverage. Recently, SNP-based platforms such as DArTseq and GBS rapidly became the standard due to their higher resolution and throughput. This transition reflects broader advances in genotyping technologies and the growing need for dense genomic data in modern bean breeding programs.

### 3.6. Genetic Diversity Metrics

Despite heterogeneity in marker systems, sample sizes, and analytical approaches, reported genetic diversity indices were summarized at the country level (Table 1). Core indices (He, Ho, PIC, FST, AMOVA) were extracted from the included studies. Their values were presented as ranges to reflect reported variability when there was more than one study in the country. No recalculation, pooling, or imputation of values was performed.

**Figure 4:**
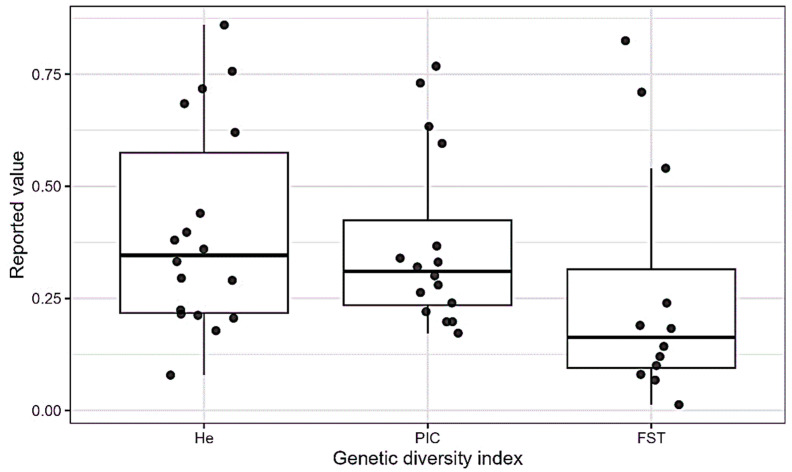
Distribution of reported genetic diversity indices (He, PIC, and FST) across the included studies. Values are presented as reported in the original publications, without recalculation or pooling.

He values covered a wide range, from low estimates in studies employing protein or allozyme markers to higher values reported in studies using SSR, SNP, and DArTseq platforms (Table 1; Figure 4). Observed heterozygosity (Ho) values were generally lower than He. The magnitude of the difference varied among countries and market systems. PIC, where reported, also exhibited broad variation, reflecting differences in marker informativeness and germplasm composition across studies (Figure 4).

**Figure 5:**
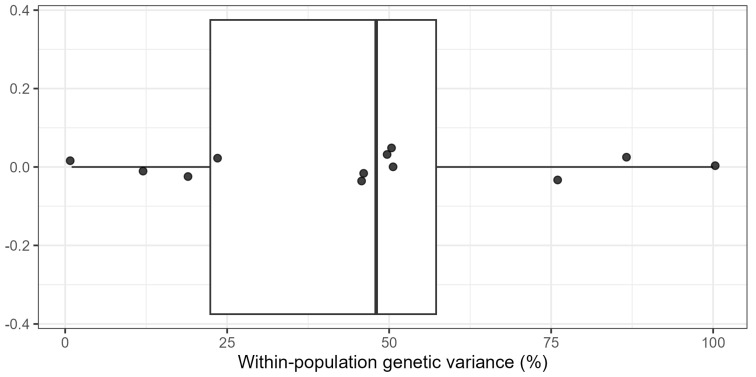
Distribution of within-population genetic variance (%) estimated by AMOVA across the included studies, as reported in the original publications.

Population differentiation, as measured by FST, was inconsistently reported across countries. However, where available, FST values generally fell within low to moderate ranges (Table 1; Figure 4). AMOVA results indicated that genetic variation was frequently attributed to within-population components rather than among-population differentiation. The reported magnitude varied across studies and countries (Figure 5). These findings highlighted the variability of reported genetic diversity despite the incomparable genetic diversity metrics across the African common bean literature.

### 3.7. Breeding and Conservation Themes

Findings pointed to four continent-wide priorities (Table 2). These are preserving unique alleles in farmer landraces through stronger gene banks, leveraging private alleles for breeding traits like drought tolerance and pest resistance, exploring admixture hotspots such as the Great Lakes for novel genetic diversity, and embracing advanced SNP technologies to improve genetic mapping and selection.

### 3.8. Risk of Bias in Studies

Risk of bias was evaluated with ROBINS-I, applied to all 29 included studies using the extraction fields. (sampling source, molecular platform, marker counts, population-structure control, and reporting completeness). To operationalize the domains for genetic-diversity studies, we pre-specified: (i) confounding, whether population structure was modelled to avoid spurious signals; (ii) selection of participants, clarity of sampling frame (on-farm vs. genebank; multi-site coverage); (iii) classification of exposure, reproducibility and validity of marker systems (SNP/SSR/AFLP vs. legacy RAPD/biochemical assays); (iv) deviations from intended exposure, protocol transparency for genotyping and analysis; (v) missing data, reporting of marker counts, call-rate thresholds, and completeness of diversity metrics (PIC/He/Ho/FST/AMOVA); (vi) measurement of outcomes, robustness of genotyping and diversity estimation; and (vii) selection of the reported result, availability of DOIs/links and congruence between methods and reported metrics. Two reviewers made independent judgments per domain with a rule-set tied to the spreadsheet fields; disagreements were resolved by consensus. At a glance, most SNP/SSR studies showed low risk in measurement and deviations, while studies employing RAPD/ISSR/POX tended to have moderate risk for measurement/classification. Where population-structure control was not reported, confounding was rated moderate or no information. Missing-data risk rose when core metrics or marker counts were not provided. A study-level traffic-light plot for all 29 papers is provided below.

## 4. Synthesis of Results

### 4.1. Study Quality

**Figure 6:**
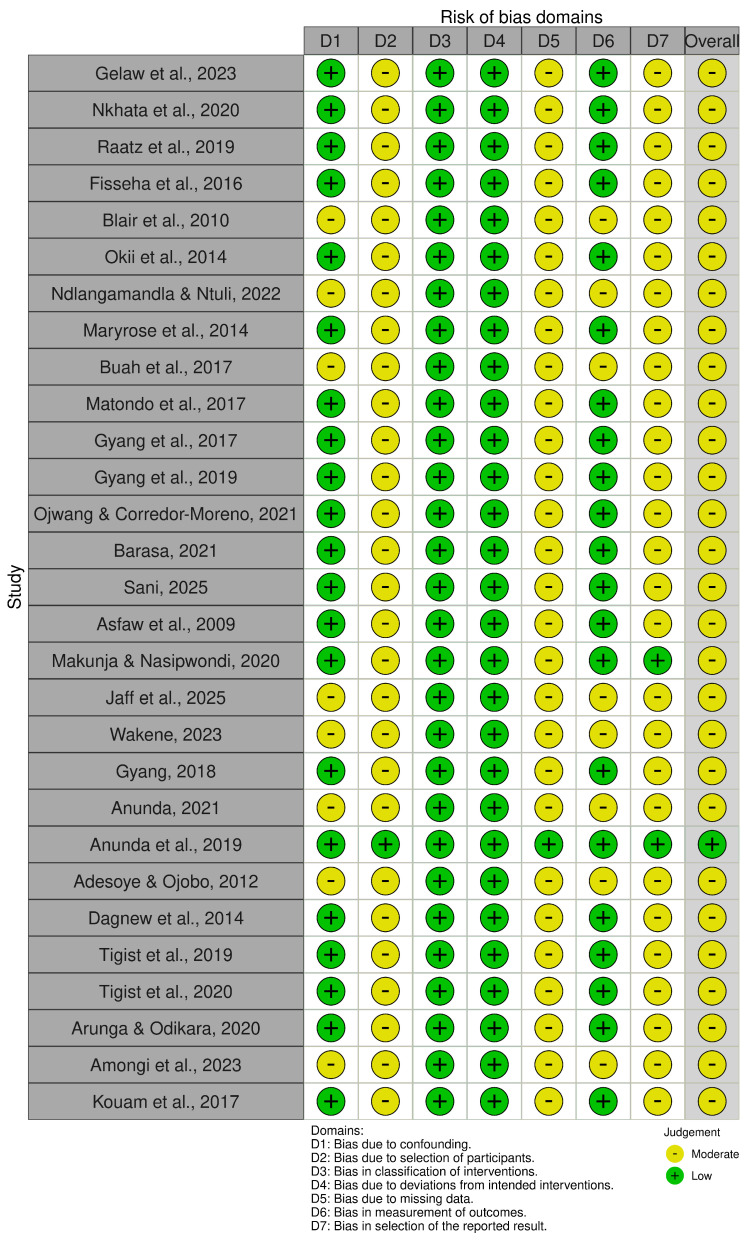
Risk of bias assessment for the studies included in this review, evaluated across do-mains related to study design, sampling strategy, reporting completeness, and methodological transparency. Most studies showed low to moderate risk across domains, with variation mainly linked to incomplete reporting of marker protocols and population structure analyses [20,21,22,24,25,26,28,42,43,44,45,46,47,48,49,50,51,52,53,54,55,56,57,58,59,60,61,62,63].

The twenty-nine investigations included in this review documented the improvement in genetic diversity studies on African common bean germplasm. A visible trend was established, from small, exploratory surveys to more extensive regional and genomic-scale analyses. That evolution was mirrored in the depth of methodological quality. Using the ROBINS-I framework, the overall evidence base fell within the low-to-moderate risk of bias range (Figure 6), illustrating how rigour in African bean research has matured over time.

Early molecular diversity studies employed microsatellite-based investigations in southern Africa [28] and in West Africa [42]. These studies worked with farmer-sourced collections that rarely exceeded a few dozen accessions and relied on a limited number of loci. These pioneering studies reported measurable polymorphism and within-population genetic variation. This was reflected by the reported PIC and He. However, these studies often omitted detailed quality-control parameters such as call-rate thresholds or complete allele-frequency tables [28,42]. As a result, uncertainty around He was relatively high. Nevertheless, these studies provided the first quantitative evidence of substantial within-population diversity in African common bean landraces.

By the mid-2010s, studies conducted in the Democratic Republic of Congo [20,21,22,43] and Nigeria [42,44] expanded both geographic coverage and experimental replication. These investigations incorporated germplasm drawn from multiple districts and national collections. This reduced selection bias and enabled clearer resolution of population structure patterns. Consequently, signals of Andean–Mesoamerican admixture that were later corroborated by higher-density marker studies were reflected in the studies. Although some aspects of reporting, such as locus-specific allele frequencies and explicit missing-data proportions, remained limited, these studies marked a clear advance in sampling design and analytical scope.

The past decade ushered in a high-density SNP era that transformed both analytical resolution and reporting transparency. Landmark studies [20,21,22] interrogated thousands of loci per accession using DArTseq or related SNP-based genotyping platforms. These studies documented quality-control procedures, including minor-allele frequency thresholds and population-structure workflows, in sufficient detail to support reproducibility and independent re-analysis. Their comparatively large sample sizes, in several cases exceeding 150 genotypes, substantially reduced sampling error and established methodological benchmarks for subsequent research.

Parallel to the expansion of high-density SNP platforms, several studies employed alternative marker systems. Some investigations used POX markers, SCoT, ISSR, phaseolin variants, and allozyme or protein markers [45,46,47,48,49,50]. Although these studies typically analysed fewer loci than SNP-based approaches, they contributed valuable complementary evidence. They also yielded information on population differentiation, allelic richness, and within-population variation across Eastern, Southern, and Central Africa. In some cases, moderate sample sizes and focused geographic sampling enabled fine-scale resolution of local diversity patterns that supported broader conclusions derived from genome-wide analyses [42,46,51].

Several investigations further strengthened inference by integrating molecular diversity data with agronomic evaluation. In South Africa [28], SSR-based genotyping was combined with replicated measurements of flowering time, pod morphology, and seed traits. Meanwhile, studies conducted in East Africa [28,51] paired SNP- or SSR-derived diversity estimates with yield-related and morphological traits. This integration enhanced the relevance of molecular findings for breeding applications. Although it required careful trait measurement protocols to minimize phenotypic bias, a standard that these studies generally satisfied.

Viewed collectively, the evidence base demonstrated a clear progression in methodological sophistication. This started with early microsatellite-based surveys of regional landraces [52,53], and more recent, large-scale SNP analyses with transparent analytical pipelines [20,21,22]. Most notable limitation was incomplete reporting of locus-specific allele frequencies in some early microsatellite studies [43,54]. However, that did not undermine the overall validity of the findings. Rather, they illustrate the gradual refinement of genetic diversity research on the African common bean. Thus, creating a literature base that is historically layered yet sufficiently robust to support continent-wide synthesis.

### 4.2. Genetic Diversity and Population Structure

Across the continent, the twenty-nine studies showed a deep allelic richness and a stable population structure. From classic microsatellite assays to thousands of single-nucleotide polymorphisms (SNPs), all showed that farmer-maintained landraces harbored wide variation within populations (Figure 5). This variation sat atop the dual ancestry of the Andean and Mesoamerican gene pools.

The magnitude of genetic diversity reported across studies was substantial. He had moderate to high ranges, while PIC values consistently indicated informative marker performance. This was consistent across all germplasm panels assembled. Studies conducted in West Africa [42,44] reported measurable PIC and He despite relatively modest sample sizes, highlighting considerable within-population variability.

A microsatellite-based study in the Democratic Republic of Congo [43] documented high He (0.6845) and PIC (0.6337), alongside low FST (0.013). The study further identified alleles unique to Congolese germplasm. These findings reinforced the role of local farmer-managed seed systems in maintaining rare genetic variants.

High-density SNP investigations [20,21,22] validated the trends established in earlier marker-based studies. This emphasised that substantial heterozygosity persists when thousands of loci are interrogated. Several of these studies reported He values exceeding 0.50 in large and diverse germplasm panels [25,26,43,47,55]. Thus, providing robust evidence for extensive genetic diversity and admixture across the continent.

Principal component and ordination analyses were largely used to unearth the gene pools in the investigations. Bayesian clustering approaches revealed widespread admixture across African common bean germplasm. In an SNP-based study [21], principal components captured a substantial proportion of genetic variation. Andean and Mesoamerican gene pools were also defined with considerable within-group variation. Comparable dual-pool patterns were reported in Southern Africa [28] and Central Africa [43]. This pattern was also evident in East African collections, where admixture reflected long-term farmer-mediated seed exchange [26,51].

Geographic comparisons reinforced these observations. East African studies in Ethiopia and Uganda [20,26] reported high He and extensive admixture. This was largely attributed to the historical seed movement across the region. Southern African panels displayed the same dual ancestry, with evidence of a stronger Andean contribution in some collections [28]. West African studies likewise identified both gene pools, although heterozygosity estimates varied among studies. The disparity reflected localized diffusion and sampling differences [42,44].

These findings paint a consistent narrative across marker systems, ecological zones, and independent studies. That is evidence enough to confer validity to a claim that common bean germplasm in Africa harbours substantial allelic diversity. Notably, there was also a stable Andean–Mesoamerican structure, which was maintained. This structure is continually reshaped by farmer seed exchange and selection, warranting breeding and conservation interventions.

### 4.3. Heterogeneity and Sensitivity

While the collective evidence painted a coherent picture of deep allelic richness and dual gene-pool ancestry, the twenty-nine studies differed markedly in sampling strategy, ecological coverage, and genotyping technology. These differences shaped the texture of the synthesis. Rather than weakening the conclusions, this heterogeneity revealed how African bean diversity expressed itself across contrasting environments and methodological eras.

#### 4.3.1. Sampling Frame and Panel Size

The size of the genetic panels varied largely across studies. As few as 10 accessions [56], to an astounding 725 genotypes were used in the investigations [51]. Smaller panels inevitably widened confidence intervals around diversity indices, thus increasing the likelihood of missing rare alleles. Larger datasets captured broader gene-pool structure [20,25,51,58,63]. West African investigations [42,44], centred on farmer landraces collected from a limited number of districts, whereas East African studies [20,26,51] drew extensively from national gene banks and regional nurseries. This dynamic sampling frame allowed the capture of both greater allelic richness and more complex population structure.

#### 4.3.2. Ecological Gradients

The study covered a wide range of agro-ecological zones. These ecological gradients likely contributed to adaptive divergence, which in some cases either paralleled or obscured underlying genetic patterns. In South Africa, overlapping landrace clusters were detected [28], even though sampling was done across distinct agro-ecological regions. This highlights the importance of incorporating environmental covariates into population-structure analyses [28].

#### 4.3.3. Marker Technology and Genotyping Depth

Methodological evolution was another major source of heterogeneity. Early studies using small suites of SSR loci and related low-density marker systems [45,52,53,54], detected broad genetic clusters. However, they provided limited resolution of fine-scale population structure. These investigations reported PIC and He indicative of moderate genetic diversity. The constraint, however was the low marker density and shallow sampling depth. In contrast, high-density SNP and DArTseq-based studies [20,21,22,51,57], interrogated thousands of markers. These studies uncovered subtle admixture patterns and rare private alleles. Thus, establishing that increased marker resolution refined inference of the same underlying genetic structure without altering its overall direction.

#### 4.3.4. Phenotypic and Trait Heterogeneity

Where agro-morphological data were integrated, the traits measured and the experimental designs varied widely. Some studies [21,28], captured flowering time, pod characteristics, and seed-related traits in replicated field trials. Meanwhile, others [44,58], focused on a narrower set of traits. It was also common for some studies [44,58], to report molecular diversity without extensive multi-season phenotypic evaluation. These inconsistencies complicated direct analysis of genotype–phenotype associations and made it practically impossible to pool trait data across regions.

#### 4.3.5. Sensitivity of the Synthesis

To assess the robustness of the conclusions, patterns reported across studies employing different marker systems, geographic scopes, and sample sizes were examined. Marker type, regional coverage, and panel size provided a comparative framework for evaluating consistency in reported outcomes. Notably high within-population genetic diversity and widespread Andean–Mesoamerican admixture were repeatedly observed. These patterns were reported in both early microsatellite-based studies and more recent high-density SNP investigations. The implication was that the consistent patterns in the studies had an utter disregard for methodological approach.

### 4.4. Reporting Bias

**Figure 7:**
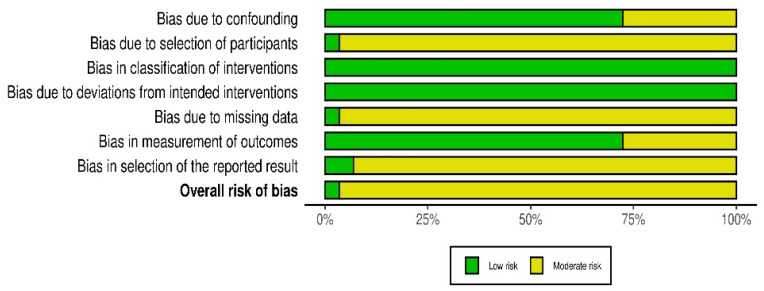
Summary plot for risk of bias.

Even with steadily improving methodological rigour, the twenty-nine studies revealed uneven transparency in how genetic and phenotypic results were documented. Applying the ROBINS-I framework to reporting practices highlighted several recurring themes (Figure 7).

#### 4.4.1. Completeness of Genetic Metrics

Several early microsatellite surveys [52,53,54], reported core diversity indices. These included PIC and He; however, they did not publish complete allele-frequency tables or detailed marker call-rate statistics. These omissions hindered independent calculation of genetic distance and fixation indices, even though the principal findings on within-population genetic variation remained credible. Mid-period studies [43,59], expanded sampling breadth and geographic coverage. However, they similarly summarised allele data without consistently providing the underlying genotype matrices required for secondary analyses.

#### 4.4.2. Population-Structure Outputs

Several studies performed principal-component or STRUCTURE/ADMIXTURE analyses but offered only text descriptions. Refs. [22,55], described cluster assignments in narrative form without publishing admixture coefficients (Q-values) or the full ordination plots needed for meta-comparisons.

#### 4.4.3. Phenotypic Trait Documentation

Where molecular data were integrated with field measurements, the trait sets and statistical reporting varied. Comprehensive, replicated protocols were followed in some studies, such as [21,28], which provided flowering time, pod metrics, and yield components with clear variance estimates. In contrast, some West African investigations recorded a narrower suite of agronomic traits and presented summary means without full dispersion statistics, limiting cross-study trait associations [42,44].

#### 4.4.4. Genotyping Pipeline Transparency

Recent SNP-based studies generally described filtering thresholds for minor allele frequency and call rate [20,21,22]. However, some studies did not explicitly report all parameters applied during SNP pruning or linkage disequilibrium filtering. This partial reporting introduced modest uncertainty when comparing heterozygosity estimates across datasets derived from different analytical pipelines.

#### 4.4.5. Accessibility and Supplementary Data

A handful of reports were published in regional outlets with limited online supplements. For example, ref. [54,56] presented sound primary analyses but provided only abbreviated data appendices, constraining independent verification.

#### 4.4.6. Synthesis of Bias Effects

Across studies, the overarching message was consistent: although specific methodological details varied, the core findings remained stable. High within-population genetic diversity and widespread Andean–Mesoamerican admixture were repeatedly confirmed across well-documented SNP- and SSR-based investigations [20,21,28,43]. Minor gaps in some early microsatellite studies or limitations in supplementary data availability, while inconvenient, did not undermine the overall findings. Accordingly, the evidence base was considered robust, with reporting bias assessed as low to moderate and unlikely to influence the main conclusions.

### 4.5. Certainty of Evidence

Across 29 studies, there was moderate-to-high confidence that African common bean germplasm was highly diverse. Moreover, there was evidence that there was a clear structure around the two classic gene pools. This conclusion was supported by three key factors: consistent genetic patterns across studies, transparent and precise analytical methods, and a low-to-moderate level of reporting bias that did not undermine the overall findings.

#### 4.5.1. Consistency of Genetic Patterns

Across sub-regions and marker systems, the principal genetic patterns were consistently recovered. High-density SNP studies reported moderate to high He and moderate levels of population differentiation. This evidences considerable within-population diversity and structured admixture between Andean and Mesoamerican gene pools [20,21,22]. Independent SSR-based investigations likewise resolved the same dual-gene-pool structure [28,43,53,54]. These patterns remained resolute across different regions and analytical platforms. That indicated that the observed genetic structure reflected underlying evolutionary history rather than artefacts of individual datasets. These trends are illustrated by the distribution of reported diversity metrics across studies (Figure 4) and by the consistent predominance of within-population variance revealed by AMOVA (Figure 5).

#### 4.5.2. Precision and Methodological Evolution

The move from low-density microsatellite markers to genome-wide SNP genotyping steadily tightened confidence around diversity estimates. Later investigations [20,21,22,51], employed thousands of loci and documented minor-allele frequency filters, call-rate thresholds, and linkage-disequilibrium pruning. This generated highly reproducible diversity statistics. The reported estimates of He, PIC, and AMOVA partitioning were showing consistent patterns across all marker systems [20,21,22,45,46,47,48,49,50,54]. This implies that the conclusions for all the included studies were robust to marker density and analytical pipelines.

#### 4.5.3. Geographic and Sampling Breadth

The evidence base spanned East, West, Central, and Southern Africa. The studies covered panels ranging from small farmer collections of fewer than forty accessions [52,53], to multi-country datasets comprising more than one hundred and fifty genotypes [20,21,22]. Despite this wide range in scale and ecological context, the direction of reported diversity patterns was consistent. This was evidenced by AMOVA in West and East Africa showing higher than among-population variation consistently [26,44]. However, there was sheer regional misrepresentation with North Africa completely lacking in the evidence base.

#### 4.5.4. Risk of Bias and Reporting Gaps

ROBINS-I assessments indicated low to moderate risk of bias overall. Early SSR studies occasionally lacked full allele-frequency tables or detailed marker call-rate information [52,53,54]. Moreover, some later investigations provided population-structure results primarily as summary admixture plots without full underlying matrices [22,55]. Yet the replication of key findings across independent, well-documented SNP and SSR studies ensured that these reporting gaps did not materially weaken the synthesis [20,21,28,43,51].

### 4.6. Overall Judgement

The evidence strongly supported two key conclusions: African common bean populations are highly diverse within themselves, and their genetic structure reflects the classic Andean and Mesoamerican gene pools, with frequent mixing between the two. While confidence was moderate for more detailed geographic patterns and trait associations due to uneven data and methods, the overall dataset offered a solid, reliable base for breeding and conservation efforts across the continent.

## 5. Discussion

The synthesis unearthed high genetic richness, structural coherence, and clear relevance for breeding under future climatic scenarios among the germplasm. Across all sub-regions, landraces consistently maintained high within-population genetic diversity. Moreover, there was a stable dual ancestry corresponding to the Andean and Mesoamerican gene pools. These patterns were supported by studies across different molecular marker eras. This was evidenced by the distribution of PIC and He (Figure 4) and the AMOVA (Figure 5), explaining the predominance of within-population genetic variance results [20,21,28,43,53]. The recurrence of these patterns, despite marked differences in ecology, marker systems, and sampling design, makes a strong case for the resilience of the African common bean germplasm in conserving allelic richness. This means that effective parental selection can be made within local germplasm without immediate reliance on exotic introductions.

Population-structure analyses reinforced this view. Principal-component and Bayesian clustering consistently resolved the two canonical gene pools with widespread admixture. This pattern was observed in multi-country panels [21,22], in single-country surveys such as those from South Africa [28], and in West and Central African collections [43,44]. AMOVA consistently attributed a greater proportion of total genetic variation to within-population differences. This implied that the principal reservoir of diversity lies within local populations rather than between regions [26,54,58]. The consistent presence of both Andean and Mesoamerican gene pools with admixtures is valuable to breeding programs. It provides breeders with unique opportunities to exploit complementary alleles for yield stability, stress tolerance, and broad adaptation. This can be accomplished through structured crossing programmes.

The heterogeneity of sampling frames and genotyping platforms, far from weakening the conclusions, provided a natural sensitivity test. There was consensus on allelic richness and admixture between early low-resolution surveys [52,53,54], and later DArTseq/SNP datasets [20,21,22,51]. He, PIC, and population differentiation conformed to a defined pattern across studies employing diverse marker systems and sampling depths, including POX, ISSR, SCoT, and phaseolin-based analyses [45,46,47,48,49,50]. That was a testament to the robustness of the review as it embraced diverse genotyping platforms and study designs.

Several studies coupled molecular data with agro-morphological traits, thus linking genetic clusters to flowering time, seed size, and yield components [21,26,28]. These associations identified trait-rich landraces that can serve as donors for breeding programs seeking tolerance to drought, pests, and soil constraints. The continued presence of private alleles in region-specific studies from the Democratic Republic of Congo and Nigeria [43,44] drummed up the pressing need to conserve local germplasm. As commercial seed systems expand, there is a real risk that these unique genetic resources could be eroded, making timely conservation efforts increasingly critical. Moreover, the germplasm represents a resilient genetic resource that can support breeding for climate variability when appropriately conserved and systematically utilised.

## 6. Conclusions

This review showed that African common bean germplasm carries deep and resilient genetic diversity. Germplasm across the reported studies consistently expressed strong within-population variation and clear signatures of Andean–Mesoamerican ancestry. The convergence of results across diverse methodologies confirmed that African bean germplasm is both genetically rich and evolutionarily active. High levels of within-population diversity highlighted the need to support both national gene banks and farmer seed systems. This helps to maintain the allelic breadth essential for future breeding. The frequent admixture and broad variation observed across regions positioned African germplasm as powerful donors for improving drought tolerance, pest resistance, and nutritional quality. Moving forward, there is a desperate need to expand dense genotyping to underrepresented regions, particularly Northern Africa, along with the Southern, Central, and Western regions. Strengthening collaborative networks between breeders, researchers, and farming communities will also be key to turning this genetic potential into more resilient and productive bean varieties for African agriculture.

## Figures and Tables

**Table 1:** Country-wise summary of molecular marker systems and reported genetic diversity metrics for *P. vulgaris* germplasm in Africa.

Country	Marker Systems Used	He	Ho	PIC	FST	AMOVA (%)
DR Congo	SSR	0.6845	0.2399	0.6337	0.013	1
Uganda	SNP; SSR; DArTseq; Phaseolin	0.206–0.6	0.097–0.5	0.172–0.6	0.0678–0.1829	46–50
Malawi	SNP	0.38	0.45	0.22	NR	51
Rwanda	Microsatellites	0.62	0.19	NR	NR	NR
Nigeria	RAPD; SNP	0.00–0.26	NR	NR	NR	NR
Ethiopia	SSR; ISSR; SNP; DArTseq; Microsatellites	0.212–0.44	0.05–0.08	0.3–0.367	0.12–0.24	12–23.53
Kenya	SSR; POX; ISSR; DArTseq; Phaseolin; SCoT	0.1778–0.86	0.06–0.7945	0.198–0.7677	0.08	87–100
South Africa	SSR	0.36	0.01	0.32	NR	NR
Cameroon	Protein markers: Allozymes	0.079	0.007	NR	NR	NR

He = expected heterozygosity; Ho = observed heterozygosity; PIC = polymorphism information content; FST = fixation index; AMOVA = analysis of molecular variance; NR = not reported.

**Table 2:** Summary of cross-cutting themes identified across the reviewed studies, highlighting evidence on conservation priorities, breeding-relevant loci, regional admixture patterns, and the transition toward high-density SNP genotyping technologies.

Theme	Evidence from Included Studies
Conservation urgency	Farmer landraces harbour private alleles; national gene banks need strengthening.
Breeding potential	Loci linked to drought tolerance, bruchid resistance, and early maturity were identified across regions.
Admixture hotspots	The Great Lakes region consistently shows Andean–Mesoamerican admixture.
Technology shift	High-density SNP genotyping is now standard, providing finer population-structure resolution.

## Data Availability

All data supporting this review are contained within the article. Additional extracted datasets (study characteristics, diversity metrics, geocoordinates) are available from the corresponding author upon reasonable request.

## Abbreviations

The following abbreviations were used in the manuscript:

He	Expected heterozygosity (Nei’s gene diversity)
Ho	Observed heterozygosity
PIC	Polymorphism Information Content
MAF	Minor allele frequency
ONA (Na)	observed number of alleles
ENA (Ne)	Effective number of alleles
I (Shannon)	Shannon’s information index
PP%	Percentage polymorphism
NPL	Number of polymorphic loci
GD	Genetic distance
GS	Genetic similarity
JSC	Jaccard similarity coefficient
FST	Fixation index (population differentiation)
GST	Genetic differentiation coefficient (Nei’s GST)
AMOVA%	Percentage variance from AMOVA (Analysis of Molecular Variance)
K	Number of genetic clusters (STRUCTURE/ADMIXTURE)

## Appendix A

**Table A1:** Summarizes the characteristics of the studies included.

Study	Country Of Study	Sample Size	Molecular Markers Used	He	Ho	PIC	MAF	ONA	ENA	I	PP%	NPL	GD	GS	JSC	FST	GST	AMOVA%	K
[20]	Ethiopia	289	DArTSeq SNP	0.3800	0.0500	0.3000	0.2800	NR	NR	NR	NR	NR	0.4900	NR	NR	0.2400	NR	23.5300	6
[21]	Malawi	60	SNP	0.3800	0.4500	0.2200	0.2400	NR	NR	NR	NR	NR	0.2800	NR	NR	NR	NR	51	2
[22]	Uganda	708	SNP	NR	NR	NR	NR	NR	NR	NR	NR	NR	NR	NR	NR	NR	NR	NR	2
[24]	Ethiopia	116	SSR	0.2900	NR	NR	NR	1.9400	NR	0.4200	100	NR	0.3500	NR	NR	0.1200	NR	12	3
[25]	Rwanda	365	Microsatellite	0.6200	0.1900	NR	NR	10.2000	NR	NR	100	30	0.03–0.45	0.55–0.97	NR	NR	NR	NR	NR
[26]	Uganda	50	SSR, phaseolin	0.6000	0.5000	0.6000	NR	4	NR	NR	95.4500	21	NR	NR	NR	0.0678–0.1829	NR	NR	4
[28]	South Africa	38	SSR markers	0.3600	0.0100	0.3200	0.7500	3.6400	NR	NR	NR	NR	NR	0.3600	NR	NR	NR	NR	2
[42]	Nigeria	11	RAPD	0.00–0.26	NR	NR	NR	1.00–1.63	1.00–1.46	NR	0–63.49	0–40	9.22–81.09	62.78–91.19	0.68–1.00	NR	NR	NR	NR
[43]	Dr Congo	91	SSR	0.6845	0.2399	0.6337	0.4354	7	NR	NR	93.7500	12	NR	NR	NR	0.0130	NR	1	NR
[44]	Nigeria	200	SNP markers	NR	NR	NR	NR	NR	NR	NR	NR	NR	NR	NR	NR	NR	NR	NR	NR
[45]	Kenya	40	POX markers	0.2149	0.6537	0.5958	NR	1.7200	1.3530	0.3306	100	NR	0.2149	NR	0.5400	NR	NR	87	2
[46]	Kenya	51	POX	NR	NR	0.2800	NR	NR	NR	NR	NR	NR	NR	0.7200	NR	NR	NR	100	3
[47]	Kenya	51	POX markers	0.718–0.757	NR	NR	NR	6	NR	NR	NR	18	0.011–0.195	NR	NR	NR	NR	NR	NR
[48]	Ethiopia	12	ISSR markers	0.2120	NR	NR	NR	NR	NR	0.3150	NR	NR	NR	NR	NR	NR	NR	NR	NR
[49]	Kenya	46	SHP1 markers, phaseolin	0.2240	NR	0.1980	NR	2.2500	NR	NR	NR	NR	NR	NR	NR	NR	NR	NR	NR
[50]	Cameroon	34	Allozymes	0.0790	0.0070	NR	NR	1.2630	NR	NR	26.3200	5	0.0650	NR	NR	NR	0.8250	NR	NR
[51]	Uganda	725	DArTseq	0.206–0.332	0.097–0.064	0.172–0.263	0–1	NR	NR	NR	NR	NR	NR	NR	NR	0.54–0.71	NR	46–50	2–11
[52]	Kenya	35	SSR	0.1778	NR	NR	NR	1.5100	NR	0.2700	51.9600	49	0.6400	NR	NR	0.0800	NR	8	9
[53]	Kenya	40	SSR markers	0.2410	NR	0.3310	NR	2.6700	1.3920	0.3330	100	3	NR	NR	NR	NR	NR	NR	2
[54]	Ethiopia	49	SSR	0.2950	0.0700	0.3670	NR	0.2630	2.7000	NR	NR	27	NR	NR	NR	NR	0.1000	NR	NR
[55]	Kenya	30	SCoT	0.8600	NR	0.7300	NR	13.1800	0	1.2800	95	NR	0.5200	NR	NR	NR	NR	4	3
[56]	Cameroon	10	Protein markers	NR	NR	NR	NR	61	NR	NR	32.9000	20	NR	0.5700	0.1430	NR	NR	NR	2
[57]	Kenya	276	DArTSeq SNP	0.3000	0.0600	0.2400	0.7900	NR	NR	NR	NR	NR	NR	NR	NR	NR	NR	NR	2
[58]	Kenya	11	Phaseolin gene	NR	NR	NR	NR	1.3400	1.2400	NR	34.4000	21.6700	0.1420	NR	0.8800	NR	NR	NR	NR
[59]	Ethiopia	192	Microsatellites	NR	NR	NR	NR	NR	NR	NR	76	231	0.03–0.45	0.55–0.97	NR	0.1900	0.1900	19	NR
[60]	Uganda	100	SSR markers	0.2990	NR	NR	NR	NR	NR	NR	71.7000	6	NR	NR	NR	NR	NR	NR	NR
[61]	Kenya	46	SSR, POX markers	0.3972	0.7945	0.7677	NR	7.2000	1.7066	0.5784	90	5	0.5840–0.6858	0.3143–0.4170	NR	NR	NR	1	7
[62]	Ethiopia	297	SNP markers	0.4400	0.0800	0.3400	NR	NR	NR	0.5800	48.7600	NR	0.6200	NR	NR	0.1200	NR	12	2
[63]	Ethiopia	297	SNP markers	NR	NR	NR	NR	NR	NR	NR	NR	NR	NR	NR	NR	NR	NR	NR	2–3
